# Imported Amoebic Liver Abscess in France

**DOI:** 10.1371/journal.pntd.0002333

**Published:** 2013-08-08

**Authors:** Hugues Cordel, Virginie Prendki, Yoann Madec, Sandrine Houze, Luc Paris, Patrice Bourée, Eric Caumes, Sophie Matheron, Olivier Bouchaud, the ALA Study Group

**Affiliations:** 1 Department of Infectious and Tropical Diseases, Avicenne Hospital, Assistance Publique-Hôpitaux de Paris and Paris 13 University, Paris, France; 2 Emerging Diseases Epidemiology Unit, Institut Pasteur, Paris, France; 3 Department of Parasitology, Bichat-Claude Bernard Hospital, Assistance Publique-Hôpitaux de Paris, Paris, France; 4 Department of Parasitology, Pitié-Salpétrière Hospital, Assistance Publique-Hôpitaux de Paris, Paris, France; 5 Department of Parasitology, Bicètre Hospital, Assistance Publique-Hôpitaux de Paris, Paris, France; 6 Department of Infectious and Tropical Diseases, Pitié-Salpétrière Hospital, Assistance Publique-Hôpitaux de Paris, Paris, France; 7 Department of Infectious and Tropical Diseases, Hôpital Bichat-Claude Bernard, Assistance Publique-Hôpitaux de Paris, Paris, France; Emory University, United States of America

## Abstract

**Background:**

Worldwide, amoebic liver abscess (ALA) can be found in individuals in non-endemic areas, especially in foreign-born travelers.

**Methods:**

We performed a retrospective analysis of ALA in patients admitted to French hospitals between 2002 and 2006. We compared imported ALA cases in European and foreign-born patients and assessed the factors associated with abscess size using a logistic regression model.

**Results:**

We investigated 90 ALA cases. Patient median age was 41. The male:female ratio was 3.5∶1. We were able to determine the origin for 75 patients: 38 were European-born and 37 foreign-born. With respect to clinical characteristics, no significant difference was observed between European and foreign-born patients except a longer lag time between the return to France after traveling abroad and the onset of symptoms for foreign-born. Factors associated with an abscess size of more than 69 mm were being male (OR = 11.25, p<0.01), aged more than 41 years old (OR = 3.63, p = 0.02) and being an immigrant (OR = 11.56, p = 0.03). Percutaneous aspiration was not based on initial abscess size but was carried out significantly more often on patients who were admitted to surgical units (OR = 10, p<0.01). The median time to abscess disappearance for 24 ALA was 7.5 months.

**Conclusions/Significance:**

In this study on imported ALA was one of the largest worldwide in terms of the number of cases included males, older patients and foreign-born patients presented with larger abscesses, suggesting that hormonal and immunological factors may be involved in ALA physiopathology. The long lag time before developing ALA after returning to a non-endemic area must be highlighted to clinicians so that they will consider *Entamoeba histolytica* as a possible pathogen of liver abscesses more often.

## Author Summary

Amœbiasis is caused by *Entamoeba histolytica (E. histolytica)*, a protozoan specific to humans which infects humans by ingestion of contaminated food and water. According to some authors, amœbiasis could be the second leading cause of death from parasitic disease worldwide. It is endemic in tropical countries but can also be diagnosed in industrialized countries, essentially in travelers. One of its main clinical manifestations is amoebic liver abscess (ALA). Abscess can be medically treated by metronidazole, but percutaneous aspiration of the abscess is sometimes performed. Few studies have been performed so far regarding the existence of cases of ALA in the industrialized world. The results of our study reported the existence of 90 cases of imported ALA in France which should raise interest among physicians as well as tourists traveling to endemic areas. Male adults and foreign-born patients presented larger abscesses, suggesting that hormonal and immunological factors could be involved in ALA physiopathology. Foreign-born patients had a longer lag time between the return to France after traveling abroad and the onset of symptoms than European-born ones. This must be highlighted to clinicians who should think about this diagnostic even if a recent travel in a tropical area is not notified by patients.

## Introduction

Amœbiasis is caused by *Entamoeba histolytica (E. histolytica)*, a protozoan specific to humans. Amœbiasis is present throughout the world, but is endemic in tropical countries where the risk of faeco-oral transmission is high [1]. The main clinical manifestations of amœbiasis are colitis and liver abscess (ALA) [1]. Pleuropulmonar and pericarditis forms also exist but ALA is the most common extraintestinal manifestation of the disease and is one of the etiologies of febrile returning travelers. In Europe, ALA is observed in European-born travelers visiting tropical countries, and in foreign-born patients living in European countries. Complications of ALA are rupture of the abscess into the neighborly cavities of the pleura, pericardium, peritoneum and distant embolic dissemination [2]. ALA was historically responsible for a high number of fatal cases, but since the introduction of medical treatment, the mortality rate is dropped to around 1 to 3%.

The management of ALA is still debated, the indications for percutaneous aspiration being a source of controversies. However most authors recognize that liver abscess puncture or drainage is generally indicated in severely ill patients, patients with large abscess, and if there is a immediate risk of rupture [1]. In France, one single study assessed treatment options for imported ALA and proposed an algorithm for drainage procedures [3]. Size of ALA is determined by radiology and is in most cases between 5 and 10 cm [2], [3], [4], [5]. Nevertheless, no factor has been identified as showing a correlation with the size of the abscess up to date.

It is critical than clinicians should be aware of the presentation, evolution and management of imported ALA.

The aim of this study was to describe the presentation of ALA in the early 2000's in Paris area where there is a high proportion of travelers and foreign born (i.e. born outside the European Union) individuals. We compared the presentation, treatment and outcome of ALA in these two populations. In addition we aimed to identify risk factors associated with abscess size at diagnosis and with percutaneous aspiration. We also aimed to describe evolution of the abscesses when follow up allowed it.

## Materials and Methods

We retrospectively investigated the medical records from patients diagnosed with ALA between January 2002 and December 2006 in 13 teaching hospitals in Paris area which agreed to participate to the study. We collected demographic data, clinical data, laboratory and radiology findings and outcome on the basis of a standardized questionnaire.

### Diagnosis of ALA

Diagnosis of ALA relied on three criteria: travel associated exposure, presence of at least one liver abscess detected by abdominal ultrasound and/or CT scan in a symptomatic patient, and a positive serology for amœbiasis.

Positive serology had to be confirmed by two of the following tests: ELISA, indirect immunoflourescence assay (IFA), indirect haemagglutination (IHA), Latex and counterimmunoelectrophoresis (CIE). Positive serology was defined when titres were above 1∶200 for ELISA, 1∶100 for IFA, 1∶320 for IHA and when there was at least one precipitation arc for CIE.

### Definition of patients' profiles and outcomes

We distinguished two populations and four patient profiles. The first population was represented by **European-born patients** defined as patients born and living in France who traveled to tropical areas (European travelers) and patients born in France living for more than 6 months in an endemic region (expatriates). The second population consisted of **foreign-born patients** defined as patients born in an endemic region, having immigrated to France who traveled back to their country of origin to visit friends and relatives (VFRs), and patients born in an endemic region who had not returned to their country of origin since they emigrated to the EU (immigrants).

We estimated time of apyrexia and biological normalization for patients who had assessment monitoring data. Abdominal ultrasounds and/or CT-scans, for those patients who had them, were reviewed to estimate the disappearance of the abscess.

### Statistical analysis

We analyzed the risk of large ALA at diagnosis, defined as a diameter greater than 69 mm, corresponding to the median abscess size in our patients, using a logistic regression model. Factors investigated were: sex of patients, age (divided into two categories defined by the median age), birthplace (France, Africa and other), patient profile (European travelers, European expatriates, Immigrants and VFR), place of contamination (Africa, Asia and other), HIV status (positive/negative), absence or presence of abdominal pain, laboratory findings such as clinically relevant haemotological and biochemical parameters : anemia defined by haemoglobin lower than 12 g/dL, a White Blood Cells count higher or lower than 10 000 per mm3, a level of C Reactive Protein higher or lower than 200, and cytolysis defined by elevated liver transaminase levels up to the normal, location of the abscess (left or right liver or unknown), number of abscesses (1, 2, 3 and 4 or more), lag time between return from an endemic area and date of the onset of symptoms, in categories based on the median (more or less than 128 days) and lag time between onset and treatment in categories based on the median (more or less than 10 days). For all of these factors, missing values were defined as “unknown”. A descriptive analysis of the dependant and independent variables was performed. A univariate analysis was then conducted, and all factors associated with large ALA having a p-value<0.20 were entered into the multivariate model. A backward stepwise selection procedure was then applied to identify significant (p<0.05) independent variables.

Logistic regression was also used to identify factors associated with the use of percutaneous aspiration. The same factors were investigated, as well as the size of the ALA (≤69 or >69 mm), the treatment and the type of care unit where the patient was admitted (*i.e.* medical or surgical department). Variables “place of birth” and “patient profile” are correlated. In our model we preferred the variable “patient profile”, which was more relevant to us because the consideration of the place of birth and reason of travelling are more appropriate for clinical practice.

Clinical profile, as well as laboratory and radiological findings were also compared between foreign-born patients (i.e., VFRs and immigrants) and European-born patients (i.e., European travelers and expatriates) using a Student t-test, chi-square test or Fischer's exact test, when required.

Using ultrasound and CT scans, we monitored the time to the disappearance of the abscess using Kaplan-Meier estimates. The Kaplan-Meier curves were compared across the different groups using the log-rank test. Factors associated with the disappearance of an abscess were identified using a Cox proportional hazard model. The factors investigated were the same as those used in the logistic regression model, and, for each factor, the proportional hazard assumption was tested based on Schoenfeld's residuals.

All analyses were performed using STATA 10.0 (Stata statistical software, Stata Corporation, College Station, Texas, USA), and a p-value≤0.05 was considered as significant.

### Ethics statement

The study received the approval of the French National Commission and Informatics and Liberties under the number 165 29 66 and all data were anonymized.

## Results

### Demographic characteristics of patients and place of acquisition (Table 1)

**Figure 1:**
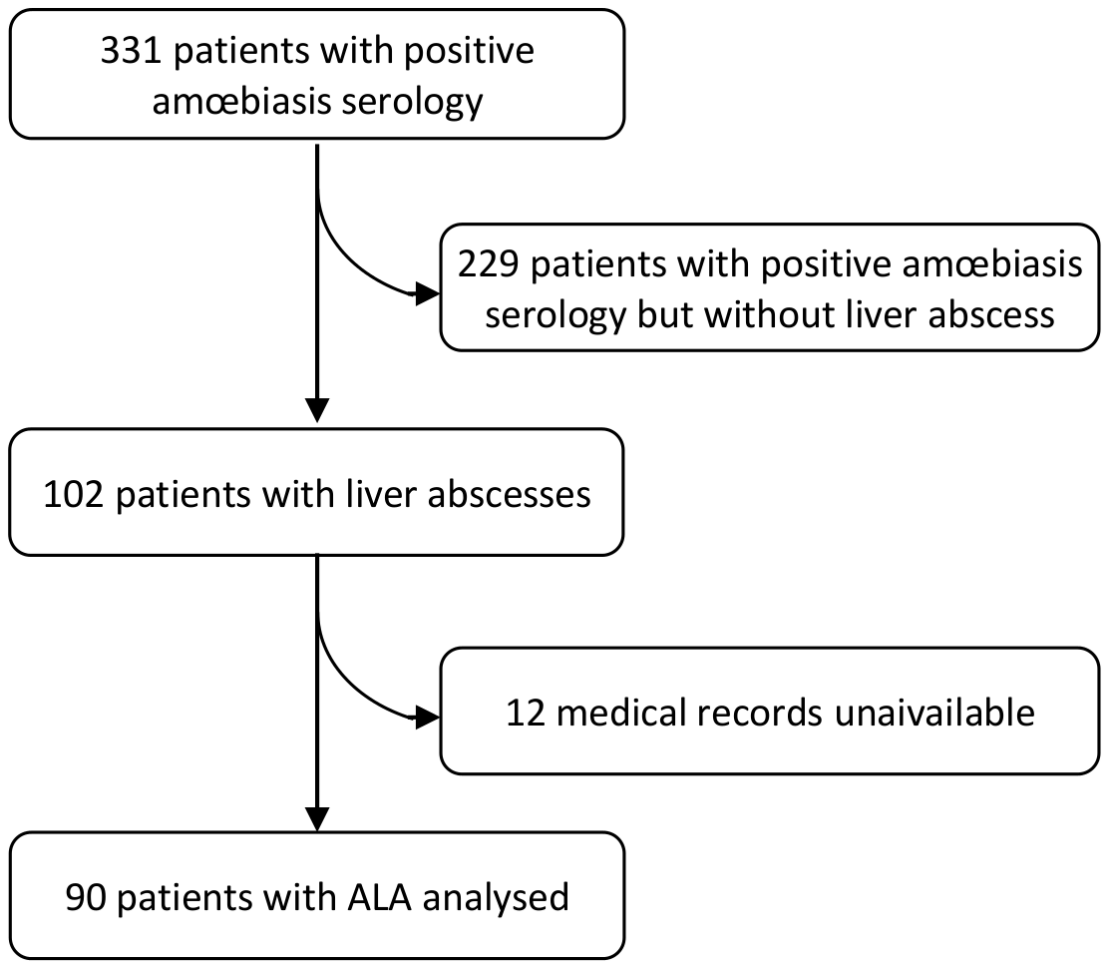
Flow chart.

331 positive amœbiasis serologies were reported. 229 positive amœbiasis serologies were without liver abscess 102 Amoebic Liver Abscesses were diagnosed during the period but 12 medical records were unaivailable (Figure 1). A total of 90 patients with ALA were identified during the study period. The male/female ratio was 3.5 and the median (inter quartile range (IQR)) age at diagnosis was 41 (33–53) years. Age distribution was not different between men and women (t-test, p = 0.46). All the patients had traveled in a region where amœbiasis is endemic: Africa (56%), Asia (19%), and South/North America (4%). There was 38 (51%) European-born patients and 37 (49%) foreign-born patients. We could not determine origin for 15 subjects. The median (IQR) time between the return from the endemic area and the first symptoms was 128 days (61–563). The median time between the first symptoms and diagnosis was 10 days (5–20) and was longer for foreign-born than European-born patients (t-test, p<0.01). Of the 79 patients tested for HIV, 4 (5%) were HIV-positive.

### Clinical, laboratory and radiology findings

Clinical and laboratory findings are summarized in Table 1. Stool sample examination was performed in 58 patients (64%) (one sample for 34 (38%) and two or more for 23 (26%)). Stool samples were performed before treatment in 11 patients and were found to be positive for *Entamoeba* cysts in five patients.

**Table 1:** Main characteristics for 90 patients with amoebic liver abscess.

		No. (%)
Sex ratio (M∶F)		3.5∶1
Age (years)	≤30	16 (18)
	31–40	26 (29)
	41–50	23 (26)
	>50	25 (28)
Place of birth	France	41 (46)
	Africa	30 (33)
	Other	11 (12)
	unknown	8 (9)
Place of contamination	Africa	50 (56)
	Asia	17 (19)
	Other	5 (5)
	unknown	18 (20)
Patient profile	European travellers °	30 (33)
	European Expatriates°°	8 (9)
	Immigrants°°°	16 (18)
	VFR°°°°	21 (23)
	unknown	15 (17)
Clinical manifestation	fever	82 (91)
	abdominal pain	73 (81)
	diarrhea	25 (28)
	nausea/vomiting	18 (20)
	jaundice	2 (2)
Laboratory Results	WBC*>10000/mm^3^	69 (77)
	haemoglobin<12 g/dL	34 (38)
	CRP**>200	59 (25)
	SGOT and/or SGPT>2N	25 (28)
	bilirubin>1N	18 (20)
Number of abscesses	1	69 (77)
	≥2	21 (23)
Lag time between return to Europe and onset	≤128 days	25 (28)
	>128 days	25 (28)
	unknown	40 (44)
Size of abscess (mm)	≤50	25 (28)
	51–75	28 (31)
	76–100	20 (22)
	>100	13 (14)
	unknown	4 (4)
Location of the absecss	right liver	62 (69)
	left liver	18 (20)
	unknown	10 (11)
Complication	pleural effusion	12 (13)
	peritoneal effusion	3 (3)
	biliary tract compression	6 (7)
	biliary tract rupture	1 (1)
	colectomy for amoeboma	1 (1)
	portal thrombus	2 (2)
Percutaneous aspiration	Yes	27 (30)
	No	61 (68)
	unknown	2 (2)

° : patients born and living in France who travelled to tropical areas °° : patients born in France living in an endemic region °°° : patients born in an endemic region and living in France who traveled back to their country to visit friends and relatives °°°° : patients born in an endemic region and living in France who did not return to their country since they immigrated * : White Blood Cells, ** C-Reactive Protein.

Diagnosis of liver abscess relied on ultrasound in 30%, CT scan in 25%, and both in 45% of cases. A single abscess was detected in 77% of cases whereas two abscesses were present in 9% of cases and three or more in the remaining 14%. The median (IQR) diameter at diagnosis was 69 mm (50–290). The main abscess location was the right lobe of the liver (78%).

Pleural effusion was found in 12 patients. Reported complications included portal thrombosis, biliary tract rupture and colectomy due to amoeboma.

### Treatment and outcome

Metronidazole was the most commonly used anti-amoebic agent (94.5% of the cases) but others, such as tinidazole (n = 3) and ornidazole (n = 2), were also used. The median (IQR) time between first symptoms and treatment, known for 62 subjects, was 9 days (5–17). Treatment duration was longer than 14 days in 39 subjects (43%). Treatment was complemented by a non-absorbed anti-amoebic luminal agent (i.e., tilbroquinol-tiliquinol) in 80% of the patients.

At least one antibiotic was additionally prescribed in 59 patients (72%). Third generation cephalosporin (n = 34), or amoxicillin alone (n = 4) or amoxicillin-clavulanate (n = 22) were used. Percutaneous aspiration was associated with the medical treatment in 27 patients. No one in this population had surgical treatment.

All the patients were hospitalized. The median (IQR) time to apyrexia after treatment initiation was three days (1–4). Median time for normalization of WBC count and CRP was 5.5 days (1–30) and 15 days (9–30), respectively. Two relapses of ALA were observed, one and three years after initial treatment, respectively. One patient had not received any anti-luminal agent during the first episode and another patient received tilbroquinol-tiliquinol. There was no fatal case.

### Comparison between European-born and foreign-born patients

Our comparison (See Table S1 in appendixes) showed that there were no significant differences between the two populations except that a longer time between return from the endemic area and the onset of the first symptoms was observed for foreign-born patients: 17% of foreign-born patients presented with an ALA more than one year after returning from a tropical area where the disease is endemic, while for European-born patients, all cases of ALA were diagnosed within a year of their return (p<0.01). The maximum lag-time between the time of return from the endemic area and the onset of symptoms was 14 years in foreign-born patients and 12 months in their European-born counterparts.

### Abscess size at the time of diagnosis and percutaneous aspiration

Data were available for 86 abscesses. Table 2 shows the results of the logistic regression analysis of factors associated with abscesses larger than 69 mm at the time of diagnosis. In multivariate analysis, men compared to women and patient older than the median age of 41 were more likely to present with a larger abscess (odds ratio (OR) = 11.25, [95% confidence interval (CI): 2.42–52.29] and OR = 3.63 [95% CI : 1.26–10.48] respectively), as were immigrants when compared with the European-born travelers' group (OR = 11.56, [95% CI : 2.10–63.41]). The estimated specificity and sensibility of the model were 71% and 70% respectively.

**Table 2:** Factors associated with initial ALA size larger than 69 mm.

		N	n with abscess size >69 mm (%)	Unadjusted OR *[95% CI]*	*p*	Adjusted OR *[95% CI]*	*p*
**Sex**	Female	19	3 (16)	1		1	
	Male	67	41 (61)	8.41 *[2.23–31.71]*	*<0.01*	11.25 *[2.42–52.29]*	*<0.01*
**Age (years)**	≤41	43	17 (40)	1			
	>41	43	27 (63)	2.58 *[1.08–6.15]*	*0.03*	3.63 *[1.26–10.48]*	0.02
**Place of birth**	France	40	14 (35)	1			
	Africa	28	20 (71)	4.64 *[1.63–13.21]*			
	Other	8	4 (50)	1.86 *[0.40–8.58]*			
	unknown	10	6 (60)	2.79 *[0.67–11.55]*	*0.03*		
**Place of contamination**	Africa	48	27 (56)	1			
	Asia	15	15 (40)	0.52 *[0.16–1.69]*			
	Other	5	2 (40)	0.52 *[0.08–3.39]*			
	unknown	18	9 (50)	0.78 *[0.26–2.30]*	*0.69*		
**Patient profile**	European travellers°	30	13 (43)	1		1	
	European Expatriates°°	8	4 (50)	1.31 *[0.27–6.24]*		1.16 *[0.20–6.61]*	
	Immigrants°°°	18	15 (83)	6.54 *[1.56–27.45]*		11.56 *[2.10–63.41]*	
	VFR°°°°	16	8 (50)	1.31 *[0.39–4.42]*		1.99 *[0.47–8.39]*	
	unknown	14	4 (29)	0.52 *[0.13–2.05]*	*0.05*	0.50 *[0.11–2.30]*	*0.03*
**HIV status**	negative	71	39 (55)	1			
	positive	4	2 (50)	0.82 *[0.11–6.15]*			
	unknown	11	3 (27)	0.31 *[0.07–1.25]*	*0.26*		
**Abdominal pain**	No	15	7 (47)	1			
	Yes	71	37 (52)	1.24 *[0.41–3.79]*	*0.70*		
WBC* count (per mm3)	≤10 000	13	8 (62)	1			
	>10 000	60	32 (53)	0.71 *[0.21–2.44]*			
	unknown	13	4 (31)	0.27 *[0.05–1.41]*	*0.26*		
**Anemia**	No	33	18 (55)	1			
	Yes	25	14 (56)	1.06 *[0.37–3.01]*			
	unknown	28	12 (43)	0.62 *[0.23–1.72]*	*0.56*		
CRP **	≤200	19	12 (63)	1			
	>200	58	28 (48)	0.54 *[0.19–1.58]*			
	unknown	9	4 (44)	0.47 *[0.09–2.34]*	*0.49*		
**Cytolysis**	Yes	75	38 (51)	1			
	No	11	6 (55)	1.46 *[0.38–5.60]*	*0.58*		
**Pleural effusion**	No	74	37 (50)	1			
	Yes	12	7 (58)	1.40 *[0.41–4.81]*	*0.59*		
**Localisation**	left liver	17	9 (53)	1			
	right liver	60	30 (50)	0.89 *[0.30–2.61]*			
	unknown	9	5 (56)	1.11 *[0.22–5.63]*	*0.94*		
**Number of abscesses**	1	66	34 (52)	1			
	2	8	4 (50)	0.94 *[0.22–4.08]*			
	3	6	3 (50)	0.94 *[0.18–5.00]*			
	≥4	6	3 (50)	0.94 *[0.18–5.00]*	*0.99*		
**Lag time between onset and treatment**	≤10 days	31	14 (45)	1			
	>10 days	29	18 (62)	1.98 *[0.71–5.57]*			
	unknown	26	12 (46)	1.04 *[0.36–2.96]*	*0.36*		
**Lag time between return to Europe and onset**	≤128 days	23	8 (35)	1			
	>128 days	24	16 (67)	3.75 *[1.21–12.54]*			
	unknown	39	20 (51)	1.97 *[0.68–5.71]*	*0.09*		

Multivariate analysis for 86 patients (missing data in 4 cases). ° : patients born and living in France who travelled to tropical areas °° : patients born in France living in an endemic region °°° : patients born in an endemic region and living in France who traveled back to their country to visit friends and relatives °°°° : patients born in an endemic region and living in France who did not return to their country since they immigrate* : White Blood Cells, ** C-Reactive Protein.

The only factor significantly associated with percutaneous aspiration in multivariate analysis was the type of department where the patient was admitted: those initially admitted to a surgical unit were more likely to have percutaneous aspiration than those admitted to a medical unit (OR = 10.0, [95% CI: 2.70–37.03]) (See Table S2 in appendixes). We couldn't identify any statistical interactions which would be clinically relevant in our models.

### Outcome of the abscesses

**Figure 2:**
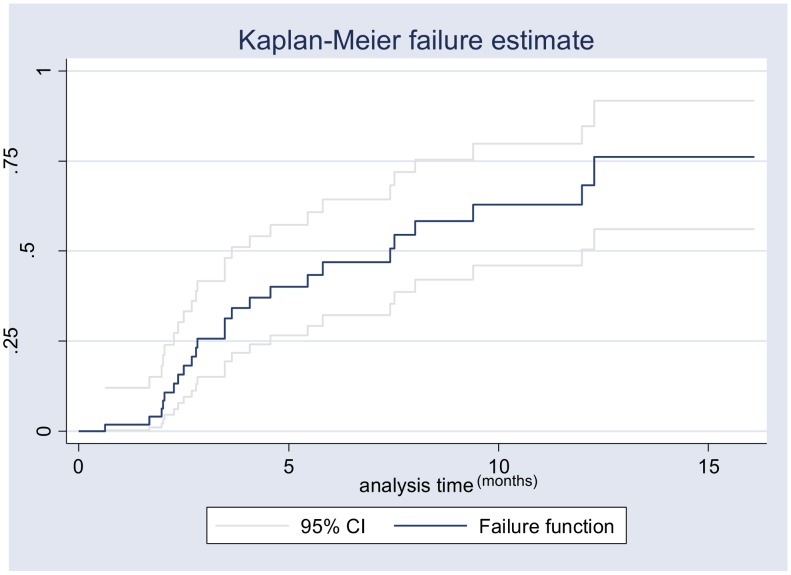
Kaplan Meier curve evaluating disappearance of ALA.

CT-Scan and/or ultrasound post-treatment monitoring was available in 24 cases. Using Kaplan Meier estimates, the observed median time to abscess disappearance was 7.5 months (Figure 2). Using a Cox proportional hazard model (See Table S3 in appendixes), the factor most strongly associated with abscess disappearance was its initial size. Larger abscesses were less likely to disappear than smaller ones: hazard ratio (HR) = 0.40 [95% CI: 0.15–1.03]; p = 0.06). Treatment (duration of antibiotherapy and percutaneous aspiration) was not associated with the probability of disappearance.

## Discussion

We identified 90 cases of ALA in the Paris area during the five-year study period. We showed that men were more likely to present with larger abscesses than women.

This is the second largest series of imported ALA. In 1984, Laverdant *et al.* reported in France 152 cases collected over 15 years in two French military hospitals [6]. In this historical and descriptive cohort, the clinical presentation was the same as in our study, with a mean abscess size of 65 mm. Nevertheless the population was different (military personnel) and percutaneous aspiration was less frequently performed than in our study (12% vs. 30%). As in other studies, ALA mainly concerns middle-aged men, presenting with fever and abdominal pain as well as elevated WBC count and CRP rate [3], [7], [8], [9], [10], [11].

ALA was much more frequently diagnosed in men than in women as in several other studies [3], [6], [8], [9], [11], [12], [13], [14]. This is unlikely that the higher proportion of men in foreign-born population living in Western countries is a relevant explanation for this. Indeed the male predominance is also found in the subgroup of European travelers (75.7% of foreign-born and 81.6% of European are male). In addition this gender tendency has already been reported for other infectious diseases [15]. For instance, analysis of pyogenic liver abscesses in Denmark over a 30-year period, showed that men were more likely to be affected [16]. This finding is also consistent with the animal model, as Lotter *et al.* demonstrated that male mice developed larger ALA than female mice [17], [18]. Hormonal factors have been suggested to explain this difference [15]. A recent study in hamsters highlights the role of sex hormones in ALA development [19]. Male hamsters, who were gonadectomized, developed either no or smaller ALAs, suggesting that testosterone could be a host factor favoring the development of ALAs. Other studies strongly support the hypothesis that immunological factors explain this gender difference. Indeed in humans, Snow et al. showed that female serum was more effective in killing *E. histolytica* trophozoïtes than a male one, thanks to the complement system [20]. In the animal model, female mice recovered more rapidly than their male counterparts due to a higher production of Interferon-γ [17] secreted by Natural Killer T Cells [18].The absence of association with HIV infection and ALA size in our study, is contradictory to this immunological hypothesis, but can be attributed once again to the low number of subjects. In a study involving more than 2000 ALA cases from an endemic region, Blessmann *et al.* showed a peak incidence at 40–49 of age as in our cohort [8]. Regarding the positive role of testosterone on susceptibility of ALA, we could expect to observe a decrease of incidence of ALA with age.

In our study, we referred to the abscess size as being the size at diagnosis, as opposed to the susceptibility to amœbiasis. For elderly patients, alteration of immunity could be determined as a factor to develop a bigger abscess than for younger patients. This could be explained by the effects of immunosenescence [21]. We have also shown that laboratory findings, especially regarding leucocytosis, C Reactive Protein and hemoglobin, were identical for large and small abscesses. This fact is to be brought to the attention to clinicians who might underestimate ALA size when expecting higher White Blood Cells count, C Reactive Protein level and more severe anemia for bigger abscess. Besides, since no significant difference was observed between the two abscess size groups regarding pleural effusion -which is one of the main complications of ALA-, it seems that localization is more to be linked to pleural complication than size itself in this case.

By comparing patients of European origin and foreign-born patients, we found a longer time between the patient's return from an endemic area and the onset of symptoms and larger abscesses in foreign-born patients. In contrast with this latter result, a study performed in the US showed that travelers born in the US were more likely to have larger abscesses (and chronic illness) compared with patients born in endemic countries [11]. In that study, immigrants came from South America and Asia whereas our patients mainly came from Africa. This longer time to develop ALA and larger ALA may both be explained by immunological reasons due to previous contact with *Entamoeba histolytica*, possibly leading to a certain protective immunity providing a less acute evolution of the disease. Although few data support this hypothesis, some studies explaining the low rate of relapse by anti-lectin IgA antibody mediated mucosal immunity [22], [23], [24] have suggested that a possible strong protective immunity develops after an episode of intestinal or liver amœbiasis. Another explanation could be that foreign-born patients have delayed access to care as this has already been described for patients infected with HIV [25], [26], a fact which led to their consulting a medical practitioner at a later disease stage. It is worth noting that among foreign-born subjects, immigrants but not VFR, seemed more likely to present with a larger abscess when compared with European-born travelers. This finding, which has not been reported in any other study may be due to better access to care for VFRs as compared to immigrants that are facing economic problems not encountered by VFRs which remain able to travel.

Nevertheless, the long delay before developing the disease, sometimes years for foreign born individuals and months for their European-born counterparts, is of clinical interest and should be highlighted. A few data are available about incubation because most of studies about ALA are performed in endemic area, where exposition is permanent. Some authors have ever suggested a long incubation, easily evaluated in case of imported ALA [5]. The problem in these studies, as in ours, is that only the last travel declared by patients is taken into account, omitting the fact they may have been contaminated during previous travel.

Clinicians should also remember that, when patients initiate treatment, apyrexia is quickly achieved, a fact which should help them to evaluate the efficiency of their treatment. In addition we were able to estimate to a median time of 7.5 months the disappearance of ALA in a quarter of the patients. This highlights the fact that recovery is a long process and that the persistence of lesions, as highlighted by radiology techniques, should not alarm clinicians during follow-up in patients free of symptoms. In addition, based on the admittedly small number of subjects in our Cox model, we found that treatment duration was not associated with any decrease in abscess size.

Percutaneous aspiration is not as effective as prolonged liver drainage but it is difficult to explain why percutaneous aspiration was not associated with any decrease in abscess size. The small number of subjects who had percutaneous aspiration monitored in our study (8 individuals) could explain these findings. Percutaneous aspiration is usually recommended for ALA bigger than 10 cm, but it is still unclear if it is really beneficial for the patient [4], [27]. In our study, patients admitted to a surgical unit were more likely to receive percutaneous aspiration. We can hypothesize that patients with a larger abscess were more likely to be admitted to a surgical unit, but our data showed that there was no difference between surgical or medical units in terms of the number of large abscesses. The greater susceptibility of surgeons for surgical treatment compared to medical treatment could explain the highest rate of percutaneous aspiration in their units.

Guidelines for the use of percutaneous aspiration do not exist. Some authors suggest that percutaneous aspiration be used for abscesses with a diameter greater than 10 cm [3], while others suggest it be performed depending on clinical evolution. Our study was not designed to answer this question.

The present study has limitations. First, the retrospective design gives rise to missing data, especially for the time between return in Europe and first symptoms. Place of contamination were unknown for 18 subjects. It could miss some autochthonous case of contamination but, comparing other series of ALA in France, this mode of contamination is exceptional. On the same hand, unknown origin (European or foreign-born) involved 15 subjects. Sensitive analyses, taking account or not these missing subjects, about factors associated to initial size of ALA had similar results. Variables with many missing values were not present in our final models.

The small number of subjects in our study is certainly responsible of a low statistical relevance. In the worst situations, an effect could have been masked in our analysis, but any false association has been shown. The sensibility and specificity of our model about abscess size were around 70%. Our study provides trends in presentation of ALA, but has been proved insufficient to bring irrefutable results.

The low number of subjects with biological and radiological data explains the low number of cases observed for the Cox multivariate model, but this original analysis is at our knowledge the first for imported cases of ALA.

Secondly, our definition of ALA was based on a positive amœbiasis serology associated to a non-bacterial liver abscess. *Entamoeba histolytica* PCR of the pus aspirate of the abscess is a very useful tool to diagnose ALA. According to case reports, serodiagnostic may lacks sensitivity when compared to PCR, as false positive serologies have been reported in endemic area [5], [28]. PCR has been evaluated in a few studies for imported ALA and should become a gold standard to diagnose ALA when invasive explorations are performed.

At least, our study collected data from 13 hospitals with different types of healthcare management. Longitudinal follow-up studies could better describe recovery and also evaluate factors associated with abscess persistence. Similarly, clinical trials are needed to assess percutaneous aspiration and to perform guidelines for its use in the treatment of ALA, something which can often be adequately treated using drugs alone.

## Conclusion

The clinical presentation and outcomes for ALA were similar in European and foreign-born patients. However foreign-born patients presented with ALA later than their Europeans-born counterparts, sometimes several years after travel. Moreover, we showed that men were more likely to present with larger abscesses than women, something which has already been observed in animal model and patients from other countries.

These data support the need for further studies on amoebic liver abscess physiopathology, including the impact of specific immunity and sexual hormones. New tools for an easier and more reliable diagnosis of ALA are also expected as in countries of imported ALA as well as in countries endemic for amœbiasis.

## Supporting Information

Table S1 **Comparison between European-born and foreign-born patients.** * : White Blood Cells, ** C-Reactive Protein.
(XLS)

Table S2 **Factors associated with percutaneous aspiration.** Multivariate analysis for 88 patients (missing data in 2 cases) ° : patients born and living in France who travelled to tropical areas. °° : patients born in France living in an endemic region. °°° : patients born in an endemic region and living in France who traveled back to their country to visit friends and relatives. °°°° :patients born in an endemic region and living in France who did not return to their country since they immigrated. * : White Blood Cells, ** C-Reactive Protein.
(XLS)

Table S3 **Cox proportional hazard model for abscess disappearance in 24 subjects.** ° : patients born and living in France who travelled to tropical areas. °° : patients born in France living in an endemic region. °°° : patients born in an endemic region and living in France who traveled back to their country to visit friends and relatives. °°°° :patients born in an endemic region and living in France who did not return to their country since they immigrated. * : White Blood Cells, ** C-Reactive Protein.
(XLS)
